# Supplementary material for: CYP26A1 Links WNT and Retinoic Acid Signaling: A Target to Differentiate ALDH+ Stem Cells in APC-Mutant CRC
Source: Cancers (Basel). 2024 Jan 7;16(2):264. doi: 10.3390/cancers16020264 (PMC10813786; doi:10.3390/cancers16020264)
Supplement: Supplementary file 1 [file cancers-16-00264-s001.zip › cancers-2800104-supplementary.pdf]

Supplementary Material

Figure S1

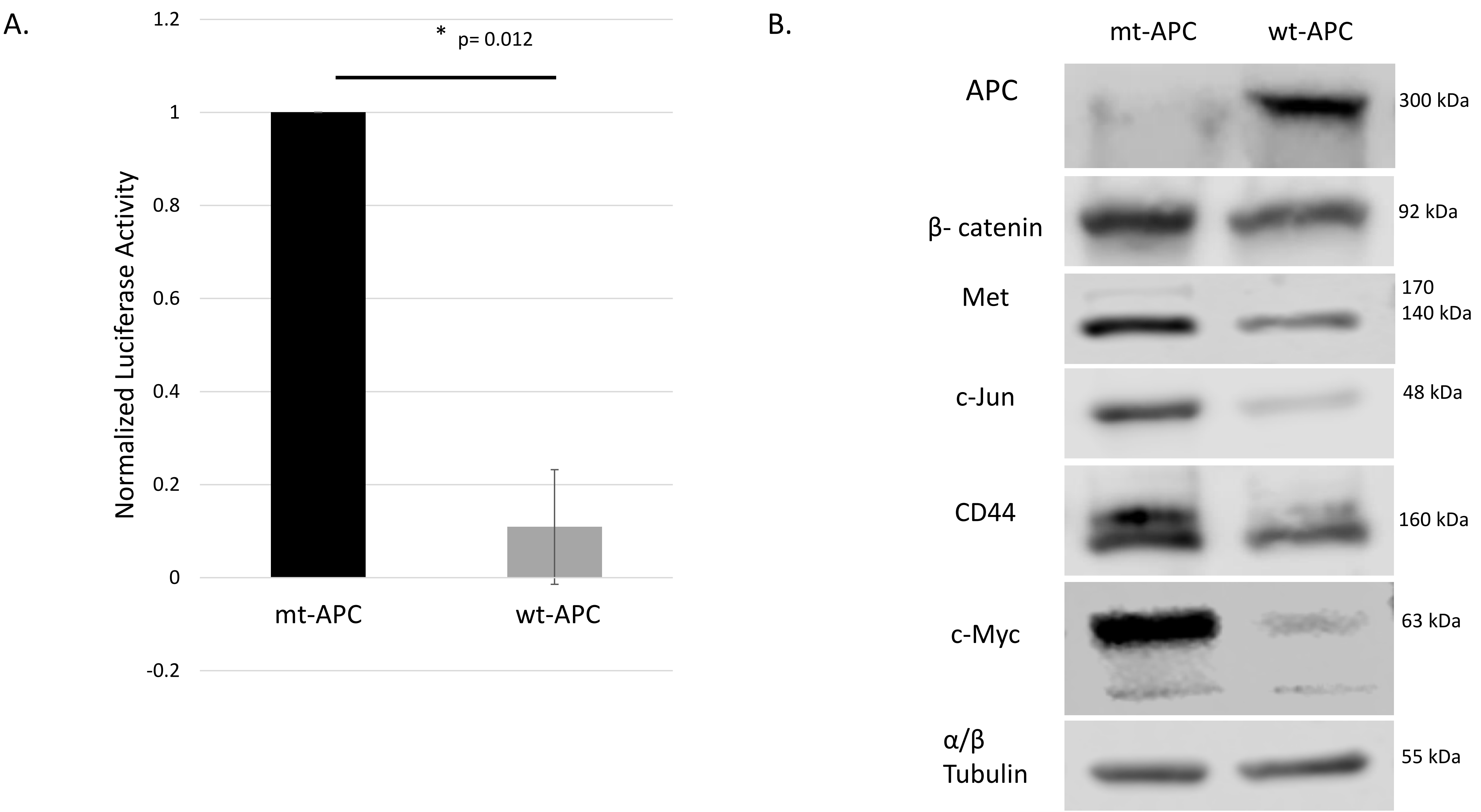

A.
1.2
1
0.8
0.6
0.4
0.2
0
-0.2
Normalized Luciferase Activity
* p= 0.012
mt-APC
wt-APC
B.
mt-APC
wt-APC
APC
300 kDa
β- catenin
92 kDa
Met
170
140 kDa
c-Jun
48 kDa
CD44
160 kDa
c-Myc
63 kDa
α/β
Tubulin
55 kDa

Figure S1. WNT/β- catenin activity is reduced with expression of *wt-APC.* WNT/β- catenin activity was measured using TCF/LEF reporter and luciferase assays. Cells were transfected with either positive or negative reporter and treated with or without zinc for 24hrs, followed by luciferase assay. (**A**) Luciferase activity was measured, and results normalized to *mt-APC*. There is a 90% decrease in WNT/ β-catenin activity when expression of *wt-APC* is induced. Student's t-test determined statistical significance as indicated. (**B**) Western blots were performed to confirm *wt-APC* expression and to assess protein expression of known WNT/TCF target proteins. Full-length, 300 kDa, APC is shown and decreased protein expression of β-catenin, MET, c-JUN, CD44, and c-MYC, are also shown.

Figure S2

A.

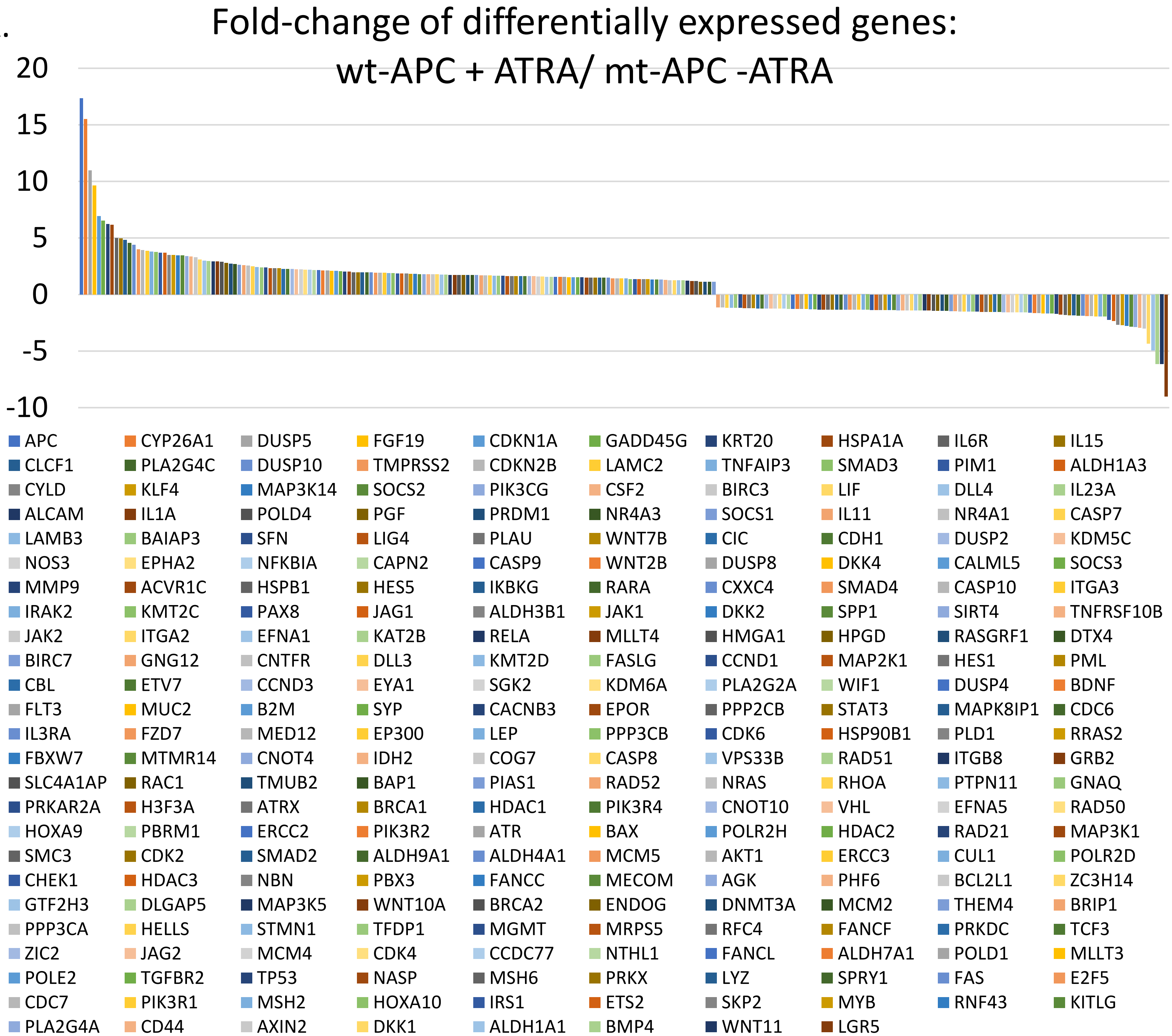
Fold-change of differentially expressed genes:
wt-APC + ATRA/ mt-APC -ATRA
20
15
10
5
0
-5
-10
APC CYP26A1 DUSP5 FGF19 CDKN1A GADD45G KRT20 HSPA1A IL6R IL15
CLCF1 PLA2G4C DUSP10 TMPRSS2 CDKN2B LAMC2 TNFAIP3 SMAD3 PIM1 ALDH1A3
CYLD KLF4 MAP3K14 SOCS2 PIK3CG CSF2 BIRC3 LIF DLL4 IL23A
ALCAM IL1A POLD4 PGF PRDM1 NR4A3 SOCS1 IL11 NR4A1 CASP7
LAMB3 BAIAP3 SFN LIG4 PLAU WNT7B CIC CDH1 DUSP2 KDM5C
NOS3 EPHA2 NFKBIA CAPN2 CASP9 WNT2B DUSP8 DKK4 CALML5 SOCS3
MMP9 ACVR1C HSPB1 HES5 IKBKG RARA CXXC4 SMAD4 CASP10 ITGA3
IRAK2 KMT2C PAX8 JAG1 ALDH3B1 JAK1 DKK2 SPP1 SIRT4 TNFRSF10B
JAK2 ITGA2 EFNA1 KAT2B RELA MLLT4 HMGA1 HPGD RASGRF1 DTX4
BIRC7 GNG12 CNTFR DLL3 KMT2D FASLG CCND1 MAP2K1 HES1 PML
CBL ETV7 CCND3 EYA1 SGK2 KDM6A PLA2G2A WIF1 DUSP4 BDNF
FLT3 MUC2 B2M SYP CACNB3 EPOR PPP2CB STAT3 MAPK8IP1 CDC6
IL3RA FZD7 MED12 EP300 LEP PPP3CB CDK6 HSP90B1 PLD1 RRAS2
FBXW7 MTMR14 CNOT4 IDH2 COG7 CASP8 VPS33B RAD51 ITGB8 GRB2
SLC4A1AP RAC1 TMUB2 BAP1 PIAS1 RAD52 NRAS RHOA PTPN11 GNAQ
PRKAR2A H3F3A ATRX BRCA1 HDAC1 PIK3R4 CNOT10 VHL EFNA5 RAD50
HOXA9 PBRM1 ERCC2 PIK3R2 ATR BAX POLR2H HDAC2 RAD21 MAP3K1
SMC3 CDK2 SMAD2 ALDH9A1 ALDH4A1 MCM5 AKT1 ERCC3 CUL1 POLR2D
CHEK1 HDAC3 NBN PBX3 FANCC MECOM AGK PHF6 BCL2L1 ZC3H14
GTF2H3 DLGAP5 MAP3K5 WNT10A BRCA2 ENDOG DNMT3A MCM2 THEM4 BRIP1
PPP3CA HELLS STMN1 TFDP1 MGMT MRPS5 RFC4 FANCF PRKDC TCF3
ZIC2 JAG2 MCM4 CDK4 CCDC77 NTHL1 FANCL ALDH7A1 POLD1 MLLT3
POLE2 TGFBR2 TP53 NASP MSH6 PRKX LYZ SPRY1 FAS E2F5
CDC7 PIK3R1 MSH2 HOXA10 IRS1 ETS2 SKP2 MYB RNF43 KITLG
PLA2G4A CD44 AXIN2 DKK1 ALDH1A1 BMP4 WNT11 LGR5

B.

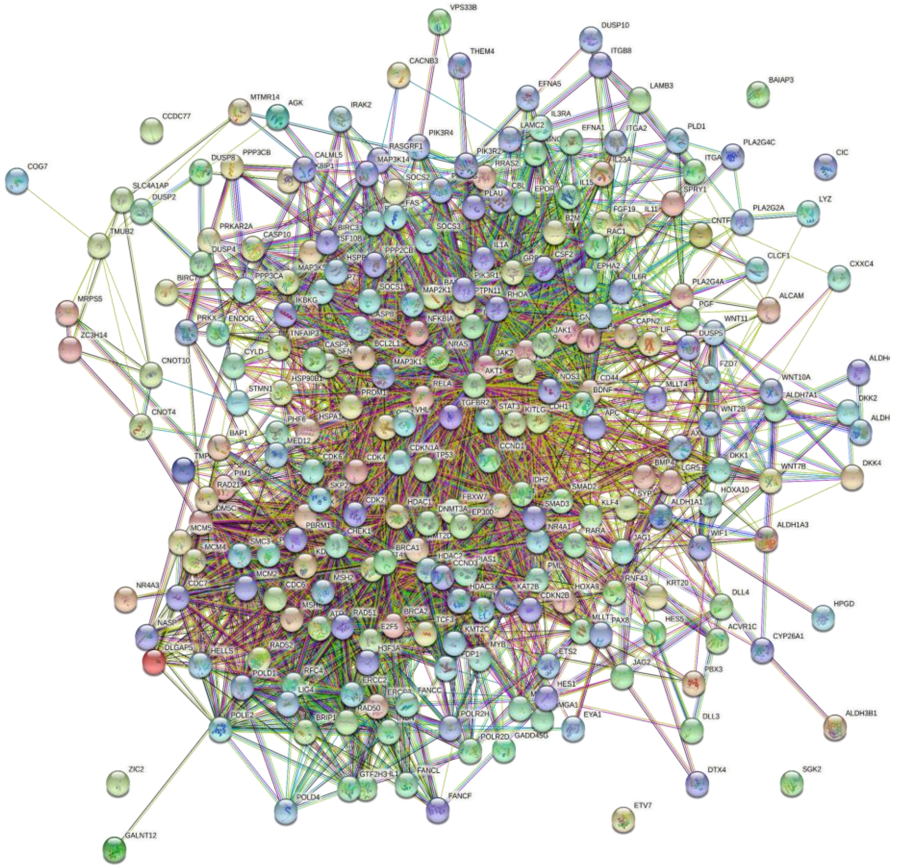

Figure S2. Inducing *wild-type APC* and treating with ATRA to drive RA signaling activates a highly regulated network of 248 proteins. HT29 cells were treated and RNA collected for NanoString profiling. Fold changes of RNA expression between treated and untreated cells were generated using nSolver software. (**A**) A list of 248- fold changes with significant *p*-values (<0.05) were selected and entered into the STRING online database. (**B**) A predicted protein network was generated, indicating a highly regulated system of protein interactions when *wild-type APC* is induced and RA signaling is activated.

Figure S3

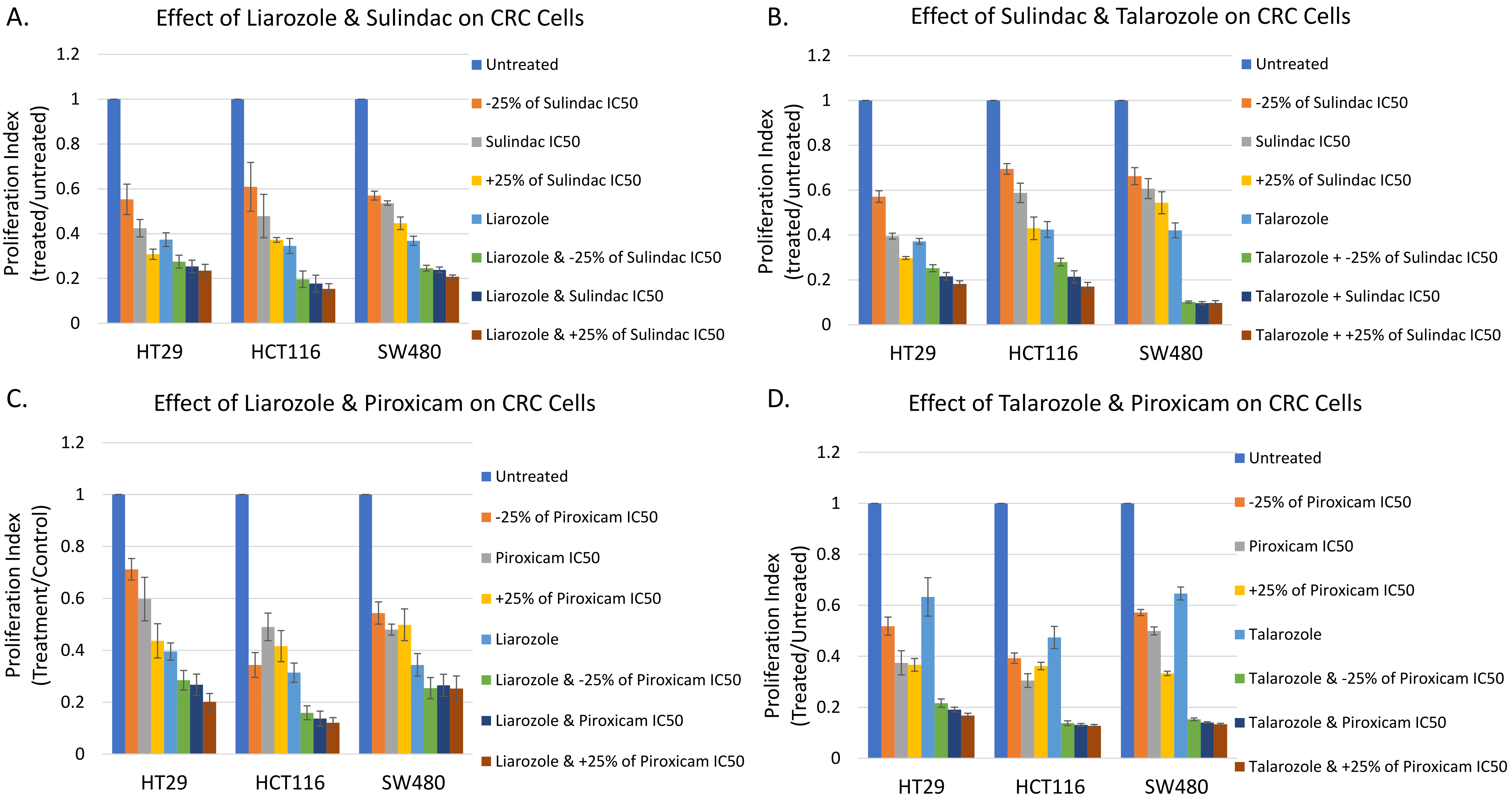
A.
Effect of Liarozole & Sulindac on CRC Cells
Proliferation Index (treated/untreated)
1.2
1
0.8
0.6
0.4
0.2
0
HT29
HCT116
SW480
Untreated
-25% of Sulindac IC50
Sulindac IC50
+25% of Sulindac IC50
Liarozole
Liarozole & -25% of Sulindac IC50
Liarozole & Sulindac IC50
Liarozole & +25% of Sulindac IC50
B.
Effect of Sulindac & Talarozole on CRC Cells
Proliferation Index (treated/untreated)
1.2
1
0.8
0.6
0.4
0.2
0
HT29
HCT116
SW480
Untreated
-25% of Sulindac IC50
Sulindac IC50
+25% of Sulindac IC50
Talarozole
Talarozole + -25% of Sulindac IC50
Talarozole + Sulindac IC50
Talarozole + +25% of Sulindac IC50
C.
Effect of Liarozole & Piroxicam on CRC Cells
Proliferation Index (Treatment/Control)
1.2
1
0.8
0.6
0.4
0.2
0
HT29
HCT116
SW480
Untreated
-25% of Piroxicam IC50
Piroxicam IC50
+25% of Piroxicam IC50
Liarozole
Liarozole & -25% of Piroxicam IC50
Liarozole & Piroxicam IC50
Liarozole & +25% of Piroxicam IC50
D.
Effect of Talarozole & Piroxicam on CRC Cells
Proliferation Index (Treated/Untreated)
1.2
1
0.8
0.6
0.4
0.2
0
HT29
HCT116
SW480
Untreated
-25% of Piroxicam IC50
Piroxicam IC50
+25% of Piroxicam IC50
Talarozole
Talarozole & -25% of Piroxicam IC50
Talarozole & Piroxicam IC50
Talarozole & +25% of Piroxicam IC50

Figure S3. Extension of data showing effects of CYP26A1 and WNT inhibition on HT29, HCT116, and SW480 CRC cell lines. Cells were plated, serum-starved, and treated for 48 hrs and crystal violet assay performed, data analyzed, and plotted as shown. Cells were treated with IC50 concentrations of CYP26A1 inhibitors- Liarozole and Talarozole. WNT inhibitor concentrations are indicated as IC50 and plus (+) or minus (-) 25% of the IC50 value.

Figure S4- A

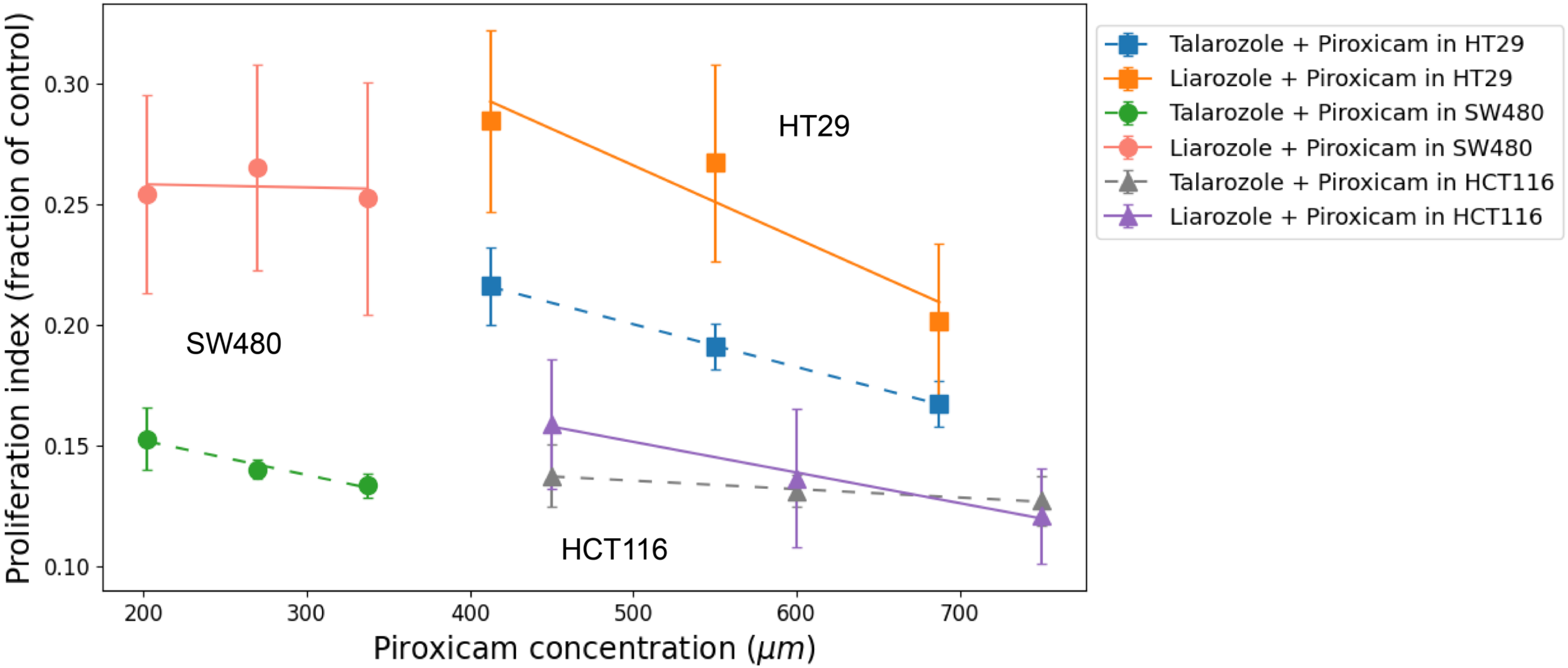

	**HT29**		**SW480**		**HCT116**	
	Slope (×10^{-5})	r^2	Slope (×10^{-5})	r^2	Slope (×10^{-5})	r^2
**Liarozole + Piroxicam**	-30.23	0.8981	-1.32	0.0164	-12.69	0.9892
**Talarozole + Piroxicam**	-17.71	0.9997	-14.22	0.971	-3.489	0.9842

Figure S4- B

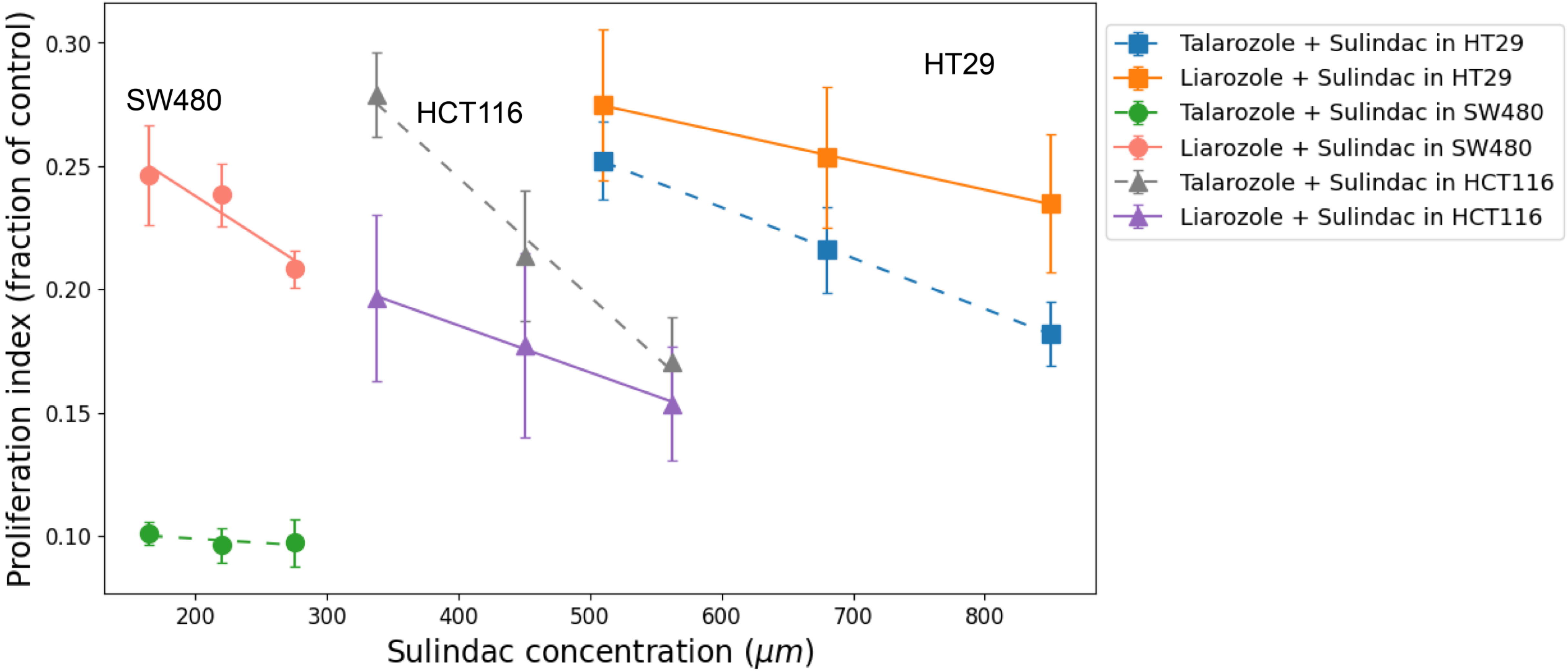

	HT29		SW480		HCT116	
	Slope (×10⁻⁵)	r²	Slope (×10⁻⁵)	r²	Slope (×10⁻⁵)	r²
**Talarozole + Sulindac**	-20.58	0.9996	-3.37	0.5468	-48.33	0.9842
**Liarozole + Sulindac**	-11.77	0.998	-34.54	0.8968	-19.07	0.997

Figure S4. Cell proliferation indexes and slopes of least-square regression plotted for combination treatments. (**A**) Slopes of combinations- Talarozole (22 to 30 μM) + Piroxicam and Liarozole (80 to 110 μM) + Piroxicam in HT29, SW480, and HCT116 cells. Piroxicam concentration was plotted along the X-axis and the proliferation index, generated via crystal violet staining, plotted on the Y-axis. Each mark represents a treatment combination with indicated drug concentrations and cell line (n = 3 replicates). Regression lines are fitted to the marks, and slopes and r^2 values indicated in table. (**B**) Slopes for combination treatments- Talarozole + Piroxicam and Liarozole + Piroxicam are plotted with Sulindac concentration along the X-axis and proliferation index along the Y-axis. Each mark represents a treatment combination with indicated drug concentrations and cell line (n = 3 replicates). Regression lines are fitted to the marks and slopes, and r^2 values are indicated in the table.

Figure S5

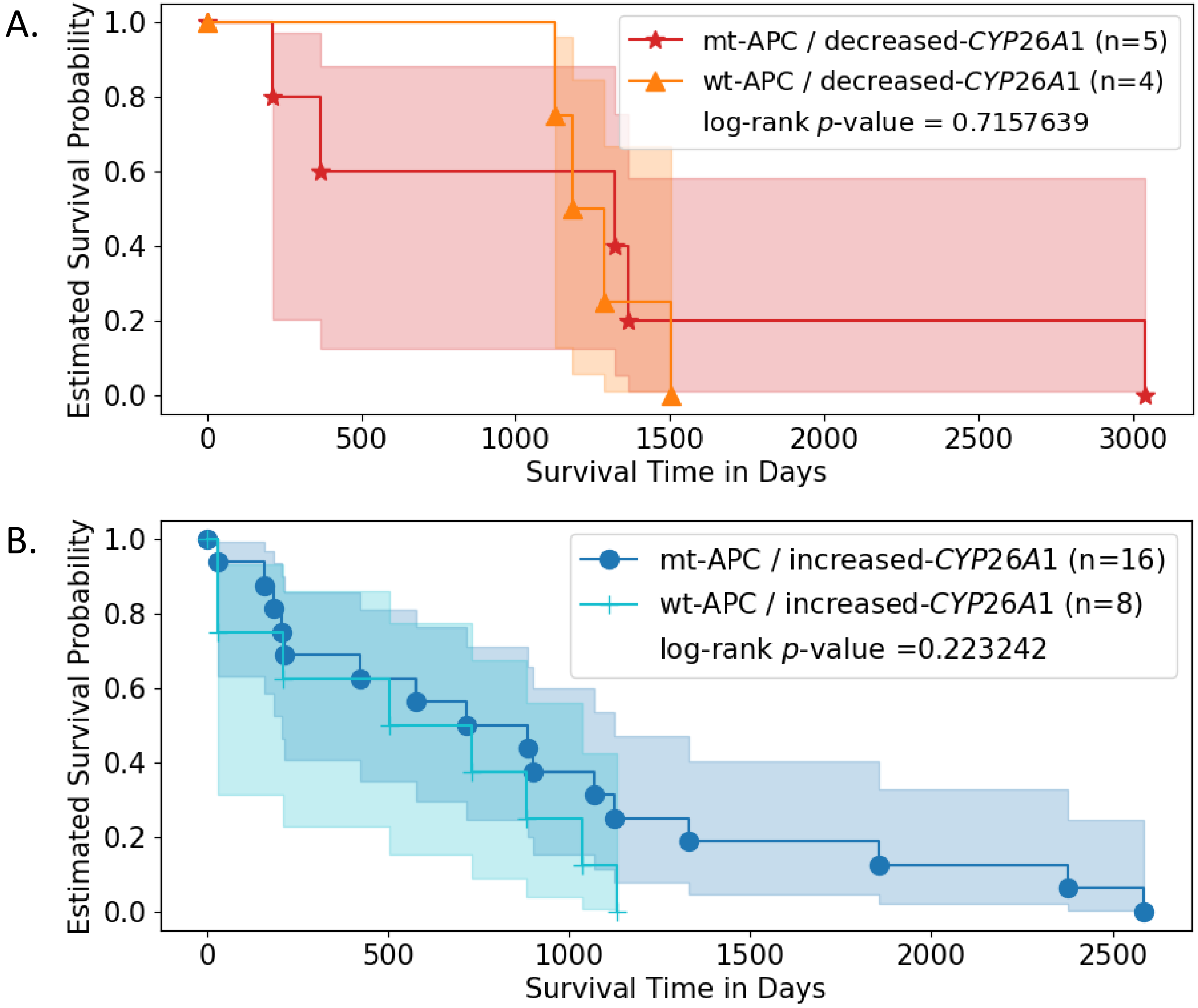
A.
mt-APC / decreased-CYP26A1 (n=5)
wt-APC / decreased-CYP26A1 (n=4)
log-rank p-value = 0.7157639
Estimated Survival Probability
1.0
0.8
0.6
0.4
0.2
0.0
0
500
1000
1500
2000
2500
3000
Survival Time in Days
B.
mt-APC / increased-CYP26A1 (n=16)
wt-APC / increased-CYP26A1 (n=8)
log-rank p-value =0.223242
Estimated Survival Probability
1.0
0.8
0.6
0.4
0.2
0.0
0
500
1000
1500
2000
2500
Survival Time in Days

Figure S5. ***APC*** **genotype is not a significant predictor of patient survival when patients are sub-grouped based on CYP26A1 level.** RNA-seq, mutation, and survival data of CRC patients were obtained from GDC data portal. Patients were grouped by *APC* mutation and change (either increase or decrease) of *CYP26A1* expression between CRC and normal colon samples from the same patient. Shaded area indicates the 95% confidence interval. (**A**) Decreased CYP26A1 expression, regardless of *APC* mutation status, is not a statistically significant predictor of patient survival. (**B**) Similarly, increased *CYP26A1* expression, regardless of *APC* mutation status, is not a significant predictor of patient survival.

Table S1

Primary Antibody	Company	Catalog #	Concentration
β- catenin	Cell Signal Technologies	8480	1:1000
Met	Cell Signal Technologies	8198	1:1000
c-Jun	Cell Signal Technologies	9165	1:1000
CD44	Cell Signal Technologies	5605	1:1000
α/β-tubulin	Cell Signal Technologies	2148	1:1000
CYP26A1	Abcam	ab172474	1:1000
CHGA	NSJ Bioreagents	RQ5334	1:1000
GLP2R	Thermo Fisher Scientific	PA5-104301	1:1000
NSE	Novus Biologicals	NB-110-58870	1:2000
SSTR1	Santa Cruz Technologies	sc-25675	1:500
APC	Millipore Sigma	OP44-100ug	1:500
**Secondary Antibody**	**Company**	**Catalog #**	**Concentration**
m-IgGkBP-HRP (anti-mouse)	Santa Cruz Technologies	sc-516102	1:1000
mouse anti-rabbit IgG-HRP	Santa Cruz Technologies	sc-2357	1:1000

Table S2

A.

CYP26A1 Inhibitor IC50 Dose (µM)		
	Liarozole	Talarozole
HT29	80	30
HCT116	110	22
SW480	80	25

B.

WNT Inhibitor Doses (µM)						
	Sulindac			Piroxicam		
	-25% IC50	IC50	+25% IC50	-25% IC50	IC50	+25% IC50
HT29	510	680	850	412.5	550	687.5
HCT116	337.5	450	562.5	450	600	750
SW480	165	220	275	202.5	270	337.5

Western Blots

Figure 3

CYP26A1

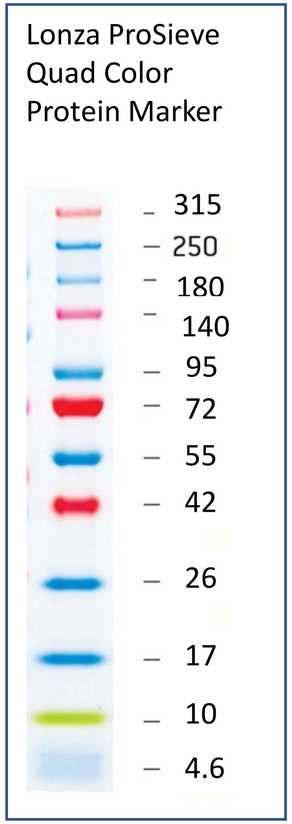

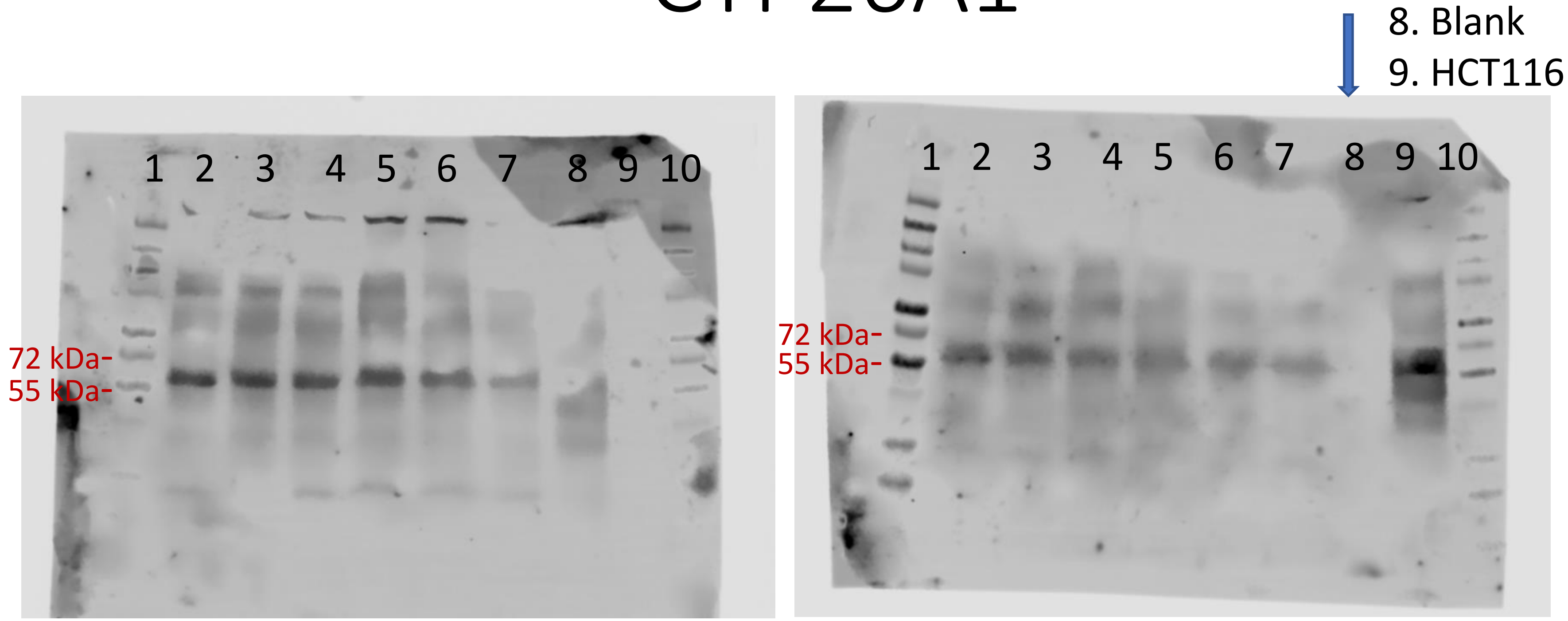

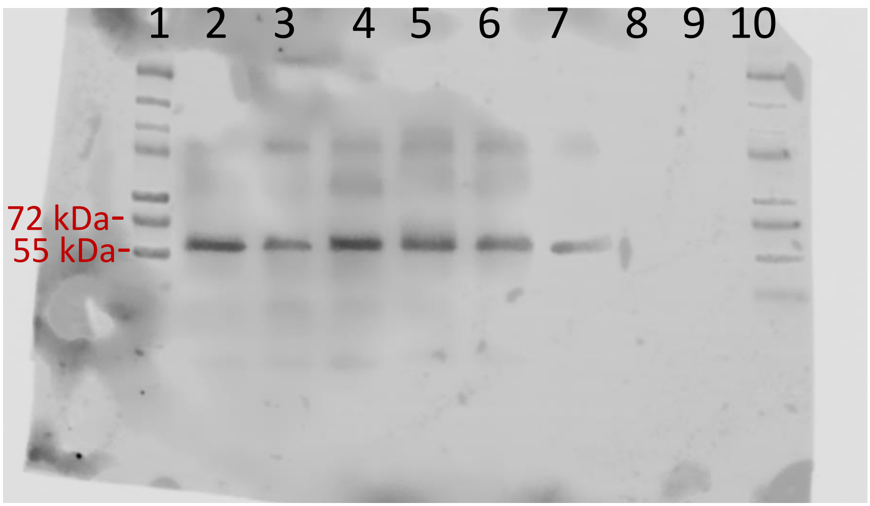

1. Protein marker
2. DMSO
3. ATRA 10
4. ATRA 20
5. DMSO
6. ATRA 10
7. ATRA 20
8. HCT116 lysate- not discussed
9. Blank
10. Protein marker

CYP26A1 Densitometry

cyp26a1	Image Name	Channel	Name	Signal	Total	Area	Bkgnd.	Type
gel 1	0000879_02	Chemi	00001	0.0702972	0.199406	2035	6.3444E-05	Signal
	0000879_02	Chemi	00002	0.0635928	0.20897	2035	7.1438E-05	Signal
	0000879_02	Chemi	00003	0.0542964	0.196611	2035	6.9933E-05	Signal
	0000879_02	Chemi	00004	0.0696345	0.198488	2035	6.3319E-05	Signal
	0000879_02	Chemi	00005	0.0432927	0.172541	2035	6.3513E-05	Signal
	0000879_02	Chemi	00006	0.0246706	0.133762	2035	5.3607E-05	Signal
gel 3	0000879_02	Chemi	00008	0.0479638	0.149997	2035	5.0139E-05	Signal
	0000879_02	Chemi	00009	0.028746	0.129758	2035	4.9637E-05	Signal
	0000879_02	Chemi	00010	0.0530092	0.168717	2035	5.6859E-05	Signal
	0000879_02	Chemi	00011	0.0469304	0.152722	2035	5.1986E-05	Signal
	0000879_02	Chemi	00012	0.0385676	0.124655	2035	4.2304E-05	Signal
	0000879_02	Chemi	00013	0.0282362	0.084056	2035	2.743E-05	Signal
gel 2	Image Name	Channel	Name	Signal	Total	Area	Bkgnd.	Type
	0000880_01	Chemi	00007	0.0227338	0.09387	1508	4.7173E-05	Signal
	0000880_01	Chemi	00008	0.0186694	0.094345	1508	5.0183E-05	Signal
	0000880_01	Chemi	00009	0.0141978	0.089194	1508	4.9732E-05	Signal
	0000880_01	Chemi	00010	0.011121	0.08638	1508	4.9907E-05	Signal
	0000880_01	Chemi	00011	0.0136076	0.078942	1508	4.3325E-05	Signal
	0000880_01	Chemi	00012	0.0098995	0.072783	1508	4.17E-05	Signal
	0000880_01	Chemi	00013	0.0325363	0.122614	1508	5.9733E-05	Signal

Figure 3

α/β-tubulin

Lonza ProSieve Quad Color Protein Marker

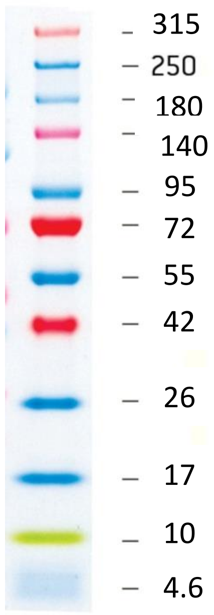

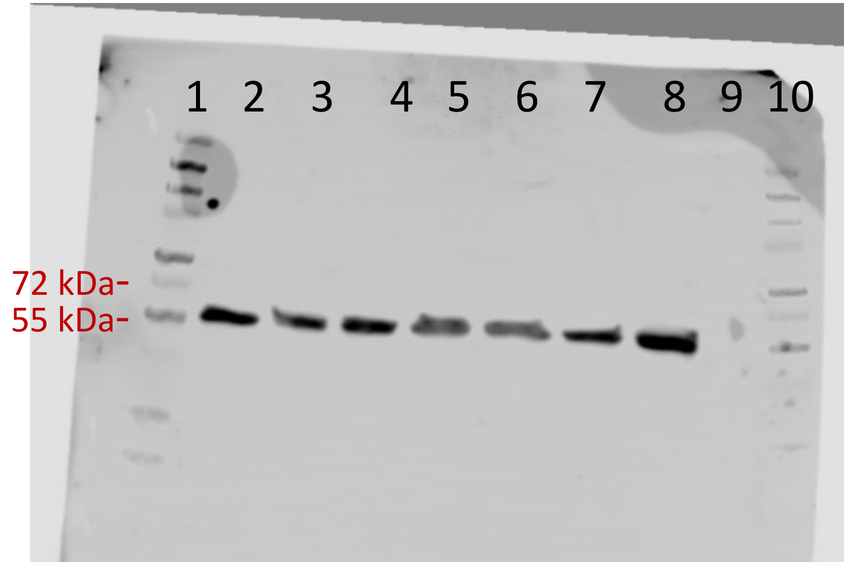

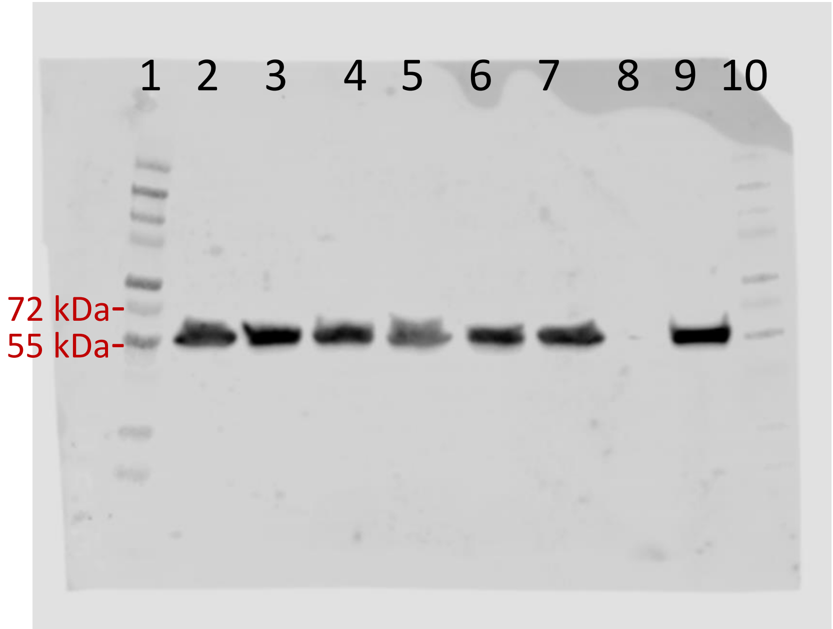

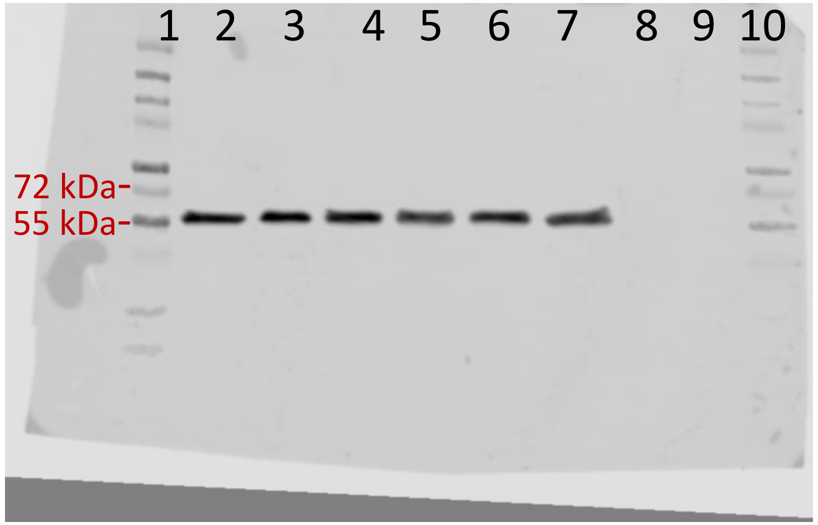

1. Protein marker
2. DMSO
3. ATRA 10
4. ATRA 20
5. DMSO
6. ATRA 10
7. ATRA 20
8. HCT116 lysate- not discussed
9. Blank
10. Protein marker

α/β tubulin Densitometry

a/b tubulin	Image Name	Channel	Name	Signal	Total	Area	Bkgnd.	Type
gel 1	0000904_02	Chemi	00001	3.9978571	4.472948	2170	0.00021894	Signal
	0000904_02	Chemi	00002	2.8248708	3.290296	2170	0.00021448	Signal
	0000904_02	Chemi	00003	3.6436223	3.970928	2170	0.00015083	Signal
	0000904_02	Chemi	00004	3.1781306	3.568618	2170	0.00017995	Signal
	0000904_02	Chemi	00005	2.7641	3.29008	2170	0.00024239	Signal
	0000904_02	Chemi	00006	3.2635473	3.586648	2170	0.00014889	Signal
	0000904_02	Chemi	00007	5.6133482	6.476317	2170	0.00039768	Signal
gel 3	0000904_02	Chemi	00008	2.8590233	3.16025	2170	0.00013881	Signal
	0000904_02	Chemi	00009	2.6162436	2.841784	2170	0.00010394	Signal
	0000904_02	Chemi	00010	3.02755	3.239487	2170	9.7667E-05	Signal
	0000904_02	Chemi	00011	2.6370391	2.836209	2170	9.1783E-05	Signal
	0000904_02	Chemi	00012	2.8342912	3.049364	2170	9.9112E-05	Signal
	0000904_02	Chemi	00013	2.8589504	3.258442	2170	0.0001841	Signal
gel 2	Image Name	Channel	Name	Signal	Total	Area	Bkgnd.	Type
	0000905_01	Chemi	00002	1.819918	2.216599	2016	0.00019677	Signal
	0000905_01	Chemi	00003	1.9979991	2.472499	2016	0.00023537	Signal
	0000905_01	Chemi	00004	1.9158432	2.196268	2016	0.0001391	Signal
	0000905_01	Chemi	00005	1.3559578	1.766118	2016	0.00020345	Signal
	0000905_01	Chemi	00006	1.7369985	1.980431	2016	0.00012075	Signal
	0000905_01	Chemi	00007	1.9097523	2.238864	2016	0.00016325	Signal
	0000905_01	Chemi	00008	2.7467265	3.022221	2016	0.00013665	Signal

Figure 5

APC- not quantified

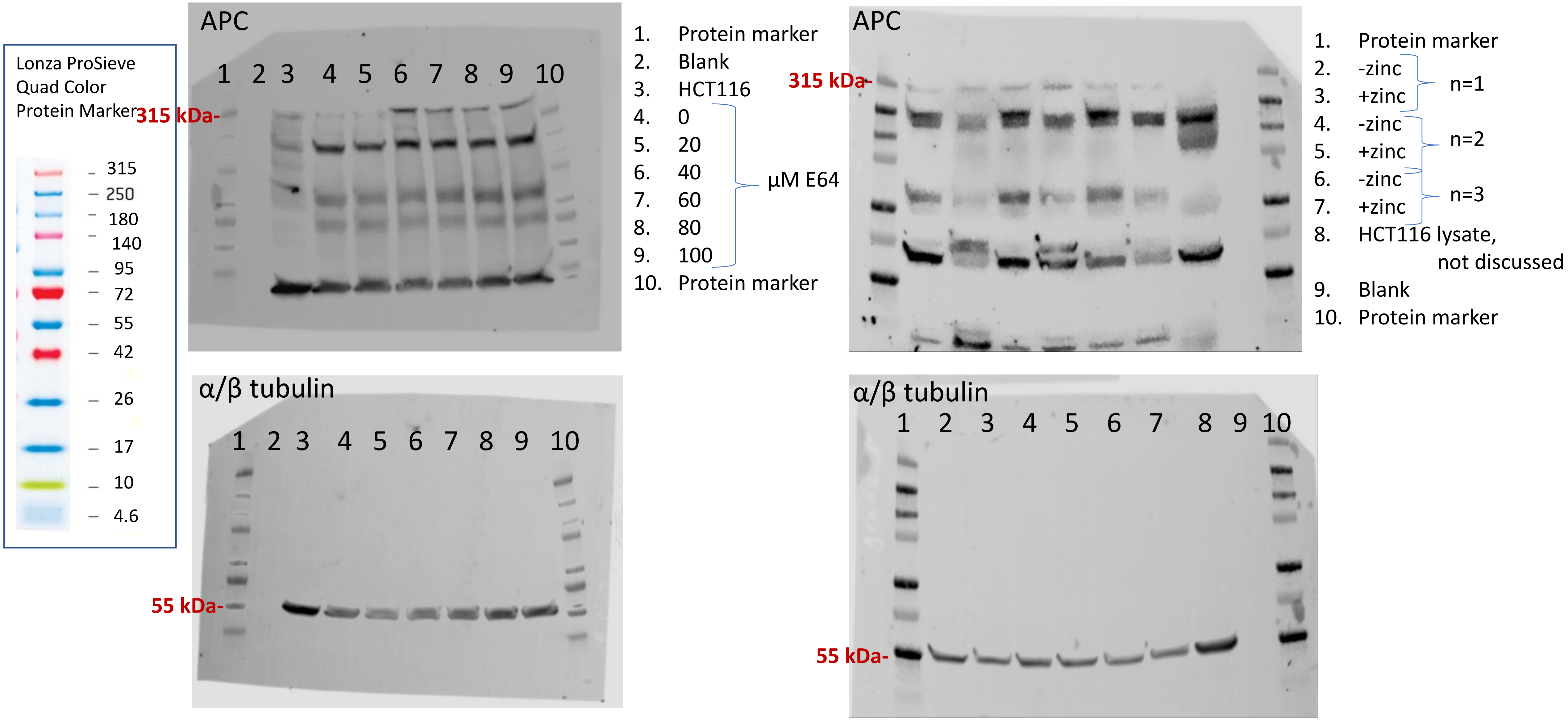

CHGA

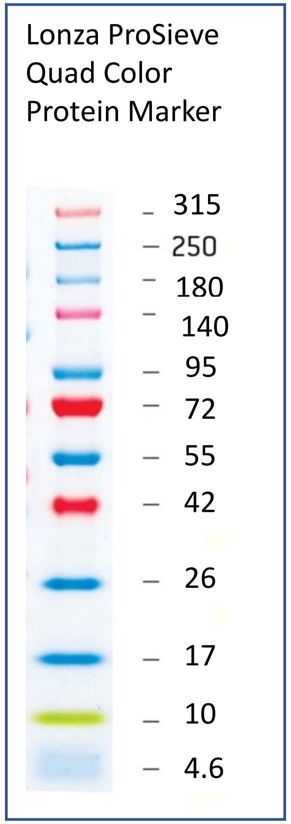

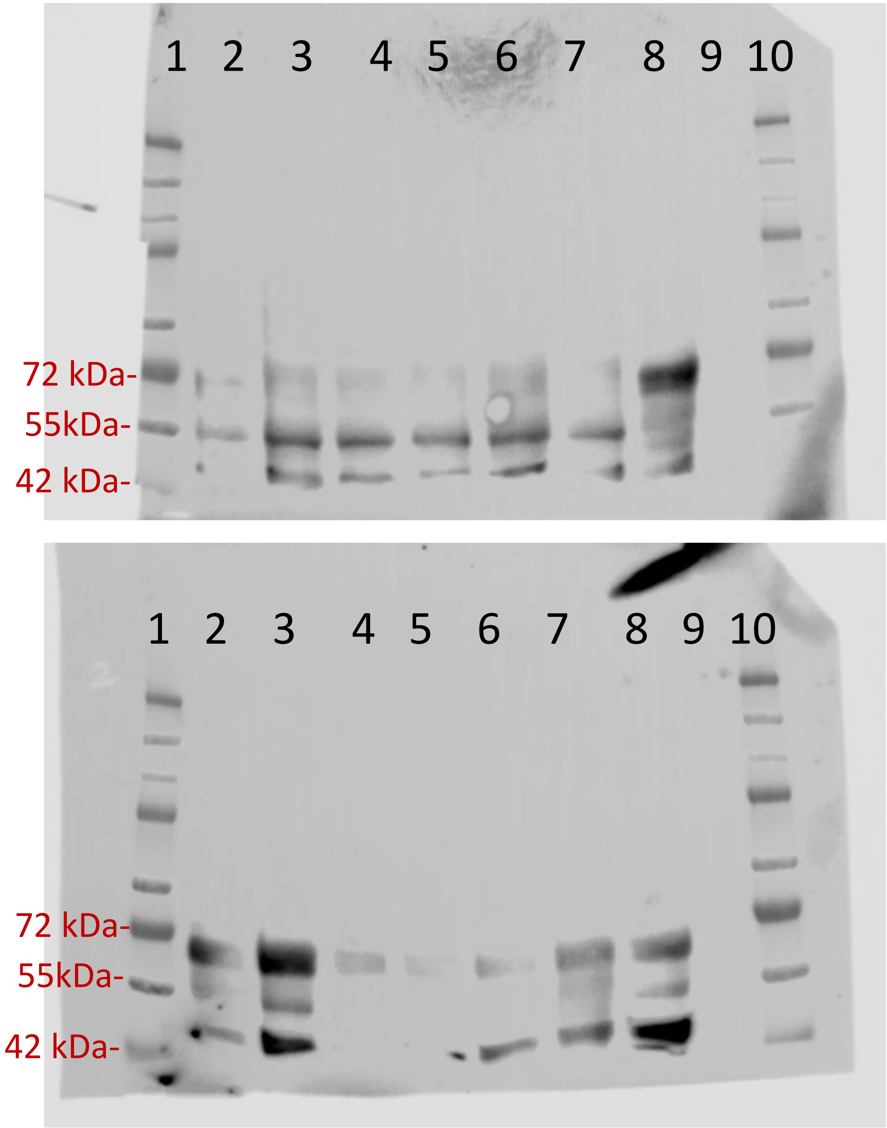

1. Protein marker
2. -zinc
3. +zinc

n=1

4. -zinc
5. +zinc

n=2

6. -zinc
7. +zinc

n=3

8. HCT116 lysate, not discussed
9. Blank
10. Protein marker

Analyzed n1 and n3

CHGA Densitometry

	CHGA	n=2 was too faint to compare.														
	Image Name	Channel	Name	Signal	Total	Area	Bkgnd.	Type	norm to tubulin	norm to c		average	no zinc	plus zinc	stdev	st err
n=3	no zinc	Chemi	00001	0.055831	0.134429	1800	4.37E-05	Signal	0.74216	1		top	1	3.529656		
	middle	Chemi	00002	0.014418	0.08745	1800	4.06E-05	Signal	0.191663	1		middle	1	4.165847		
	bottom	Chemi	00003	0.086348	0.175677	1800	4.96E-05	Signal	1.147832	1		bottom*	1	6.503912	1.222415	0.864378
	plus zinc	Chemi	00004	0.13446	0.252919	1800	6.58E-05	Signal	2.544741	3.428829						
	middle	Chemi	00005	0.0502	0.155646	1800	5.86E-05	Signal	0.950072	4.957003						
	bottom	Chemi	00006	0.342035	0.505902	1800	9.1E-05	Signal	6.47324	5.639534						
	Image Name	Channel	Name	Signal	Total	Area	Bkgnd.	Type	norm to tub	norm to control						
n=1	0000585_02	Chemi	no z top	0.130288	0.311586	2394	7.57E-05	Signal	1.253706	1						
	0000585_02	Chemi	z top	0.277455	0.480691	2394	8.49E-05	Signal	4.551558	3.630484						
	0000585_02	Chemi	no z middle	0.036226	0.206204	2394	7.1E-05	Signal	0.355272	1						
	0000585_02	Chemi	z middle	0.084889	0.271037	2394	7.78E-05	Signal	1.198932	3.37469						
	0000585_02	Chemi	no z bottom	0.035362	0.203078	2394	7.01E-05	Signal	0.346795	1						
	0000585_02	Chemi	z bottom	0.180924	0.360679	2394	7.51E-05	Signal	2.555289	7.36829						

GLP2R

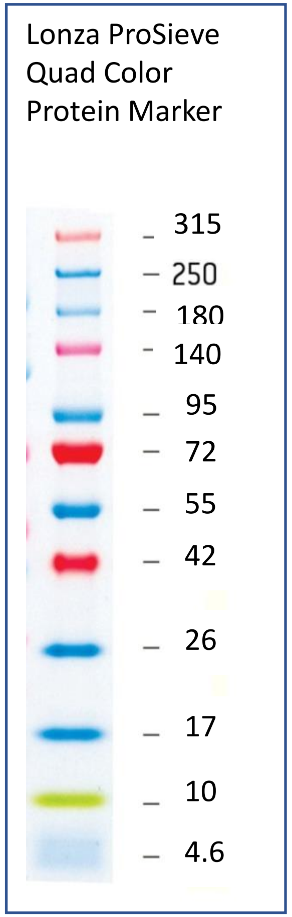

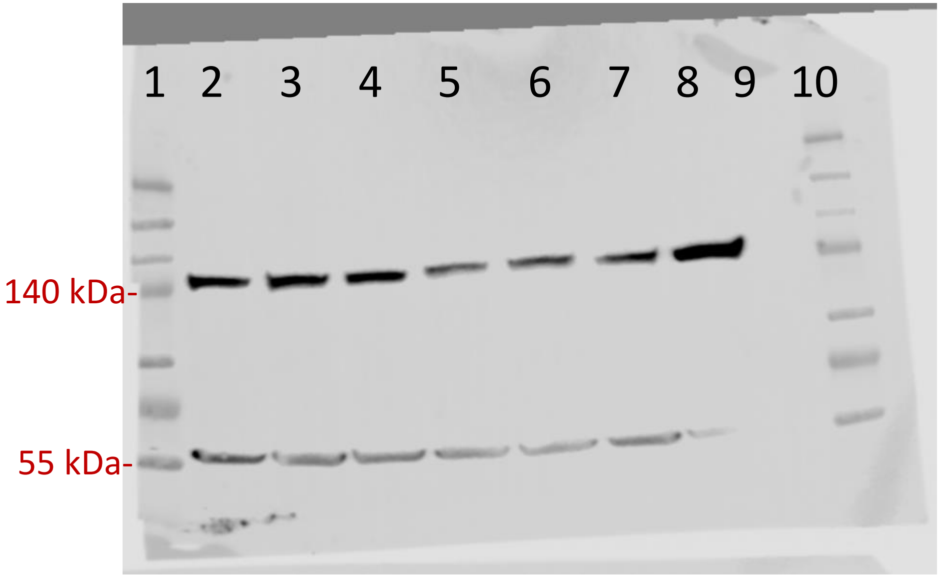

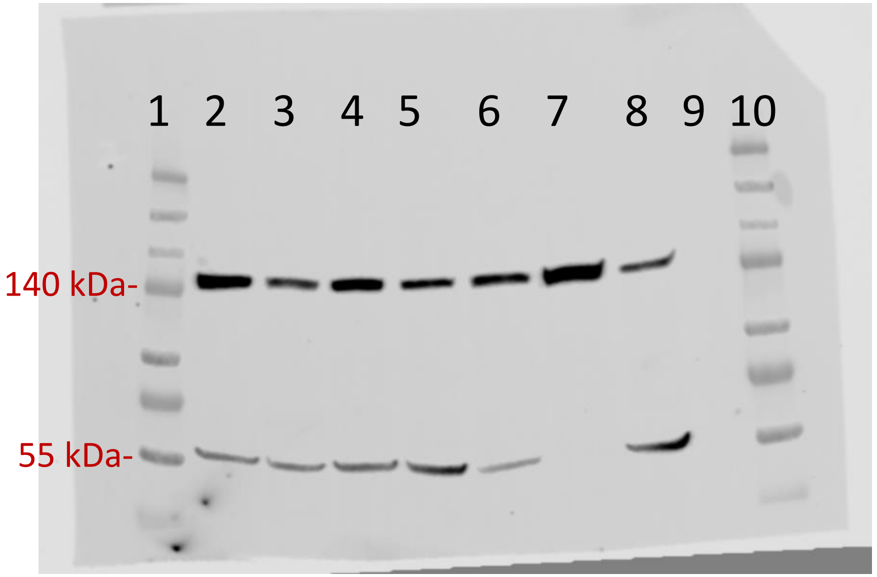

1. Protein marker
2. -zinc } n=1
3. +zinc
4. -zinc } n=2
5. +zinc
6. -zinc } n=3
7. +zinc
8. HCT116 lysate, not discussed
9. Blank
10. Protein marker

Analyzed 60 kDa band

GLP2R Densitometry

	GLP2R												
	Image Name	Channel	Name	Signal	Total	Area	Bkgnd.	Type	norm to tub	norm to control	average	st dev	st err
n=1	0000595_02	Chemi	00001	0.031794	0.040883	1728	5.26E-06	Signal	0.311805	1	2.159973	1.481436	0.855307
	0000595_02	Chemi	00002	0.026924	0.036439	1728	5.51E-06	Signal	0.380267	1.219567			
n=2	0000595_02	Chemi	00003	0.039411	0.046879	1728	4.32E-06	Signal	0.448849	1			
	0000595_02	Chemi	00004	0.048722	0.059078	1728	5.99E-06	Signal	0.625106	1.392686			
n=3	0000595_02	Chemi	00005	0.022542	0.028511	1728	3.45E-06	Signal	0.299656	1			
	0000595_02	Chemi	00006	0.061238	0.069057	1728	4.52E-06	Signal	1.158968	3.867665			

Enolase/ NSE

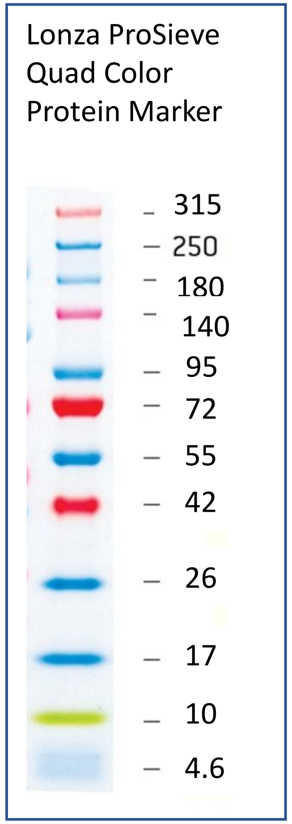

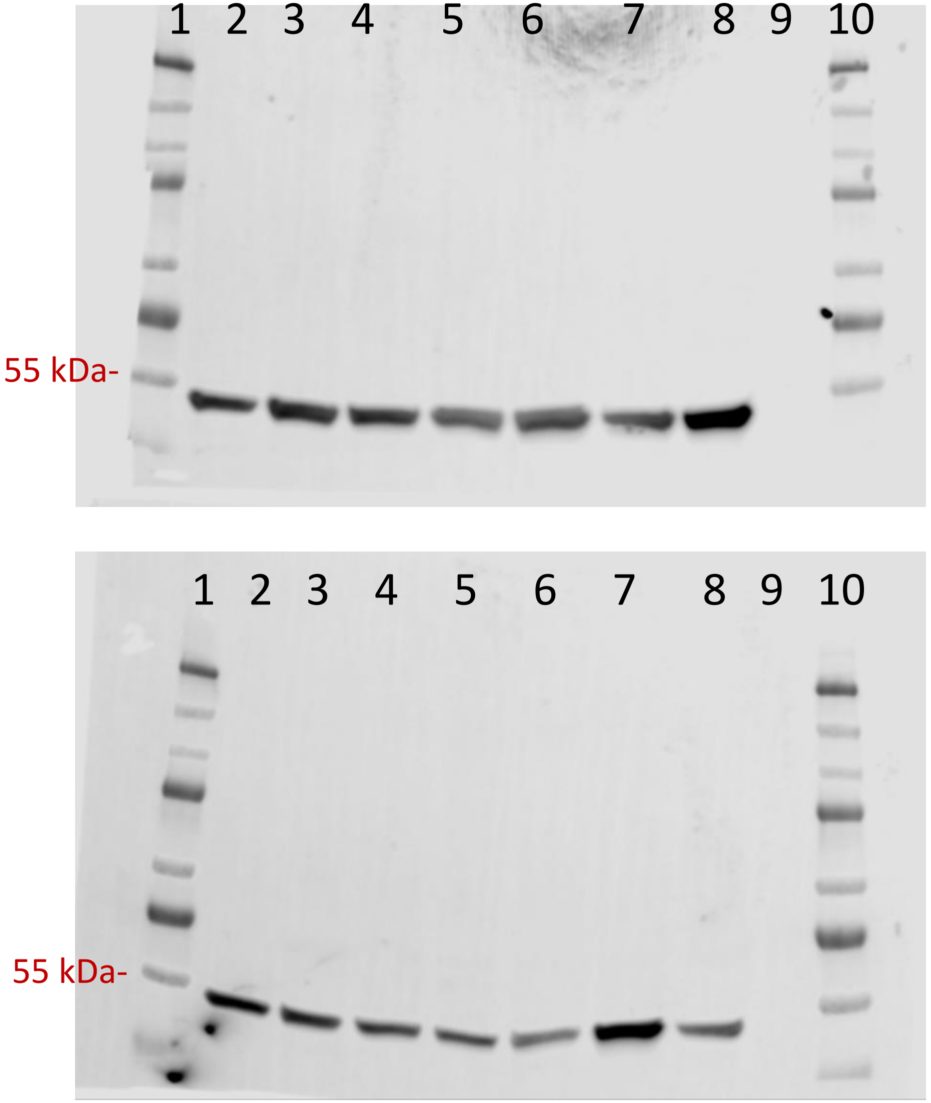

1. Protein marker
2. -zinc
3. +zinc
4. -zinc
5. +zinc
6. -zinc
7. +zinc
8. HCT116 lysate, not discussed
9. Blank
10. Protein marker

(Lanes 2–3: n=1; lanes 4–5: n=2; lanes 6–7: n=3)

NSE Densitometry

	Enolase/ NSE												
	Image Name	Channel	Name	Signal	Total	Area	Bkgnd.	Type	norm to tub	norm to control	average	st dev	st err
n=1	0000601_02	Chemi	00001	9.498009	10.29861	1920	0.000417	Signal	93.14737	1	1.308985	0.408328	0.235748
	0000601_02	Chemi	00002	7.670272	8.603663	1920	0.000486	Signal	108.3312	1.163009			
n=2	0000601_02	Chemi	00003	6.562607	7.560053	1920	0.00052	Signal	74.74105	1			
	0000601_02	Chemi	00004	5.788858	6.508143	1920	0.000375	Signal	74.27075	0.993708			
n=3	0000601_02	Chemi	00005	4.791397	5.919724	1920	0.000588	Signal	63.69249	1			
	0000601_02	Chemi	00007	5.957571	6.565863	1920	0.000317	Signal	112.7509	1.770239			

SSTR1

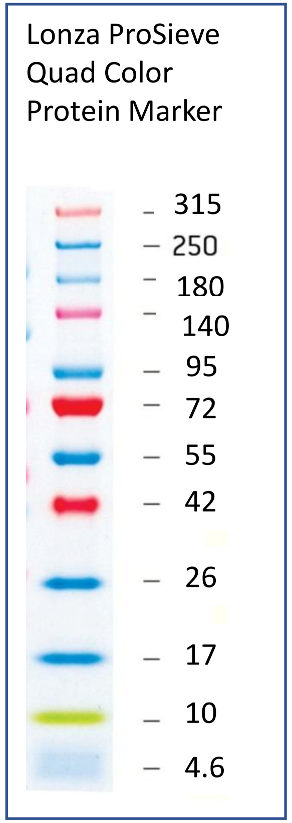

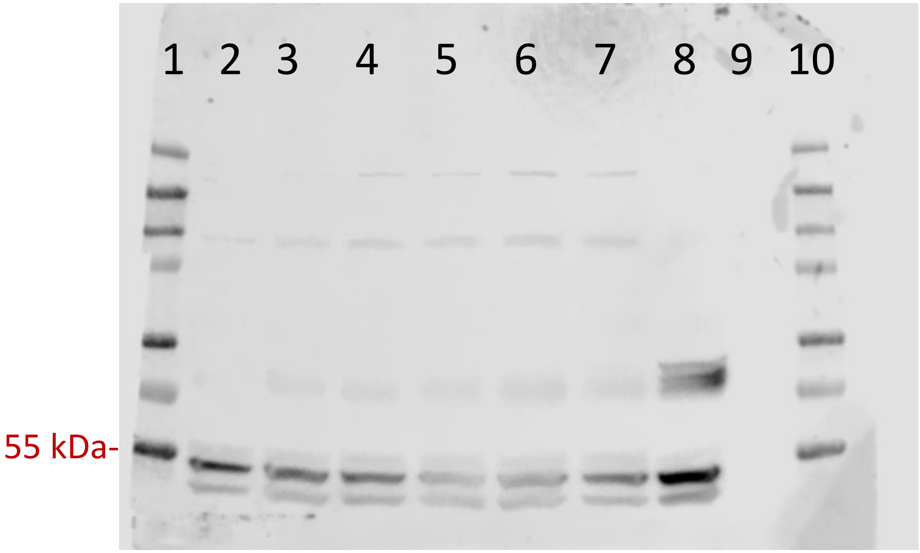

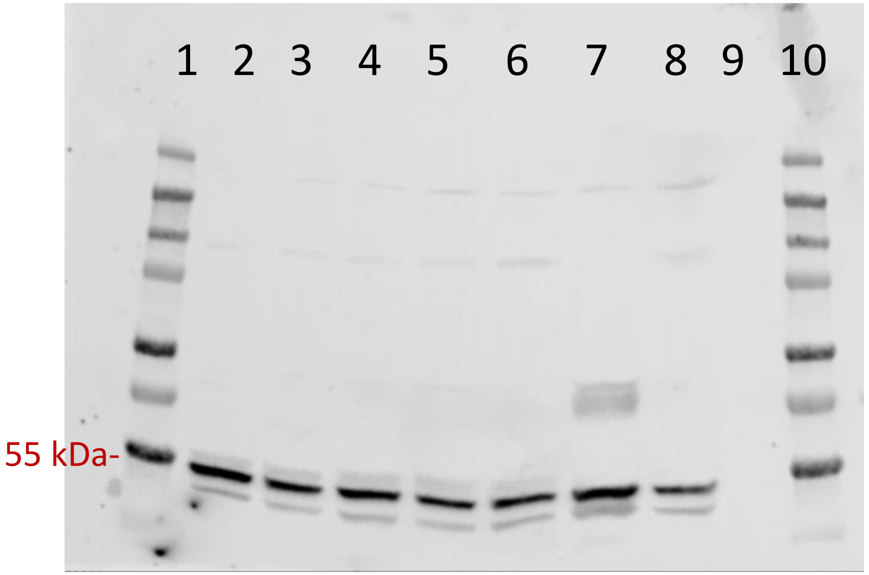

1. Protein marker
2. -zinc
3. +zinc
4. -zinc
5. +zinc
6. -zinc
7. +zinc
8. HCT116 lysate, not discussed
9. Blank
10. Protein marker

Lanes 2–3: n=1; lanes 4–5: n=2; lanes 6–7: n=3

SSTR1 Densitometry

	SSTR1													
	Image Name	Channel	Name	Signal	Total	Area	Bkgnd.	Type	norm to tub	norm to control	average	st dev	st err	
n=1	0000608_02	Chemi	00001	0.484174	0.735849	1440	0.000175	Signal	4.748314	1	1.012268	0.210822	0.121718	
	0000608_02	Chemi	00002	0.344003	0.499221	1440	0.000108	Signal	4.858538	1.023213				
n=2	0000608_02	Chemi	00003	0.400521	0.579465	1440	0.000124	Signal	4.561509	1				
	0000608_02	Chemi	00004	0.283073	0.448719	1440	0.000115	Signal	3.631809	0.796186				
n=3	0000608_02	Chemi	00005	0.306848	0.482729	1440	0.000122	Signal	4.078959	1				
	0000608_02	Chemi	00006	0.262381	0.351485	1440	6.19E-05	Signal	4.965741	1.217404				

α/β tubulin

Lonza ProSieve Quad Color Protein Marker

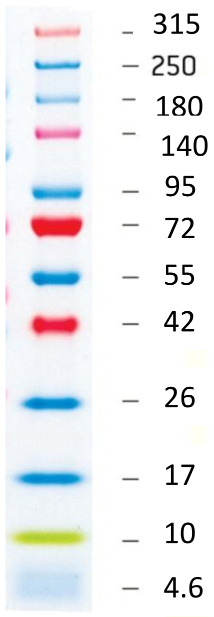

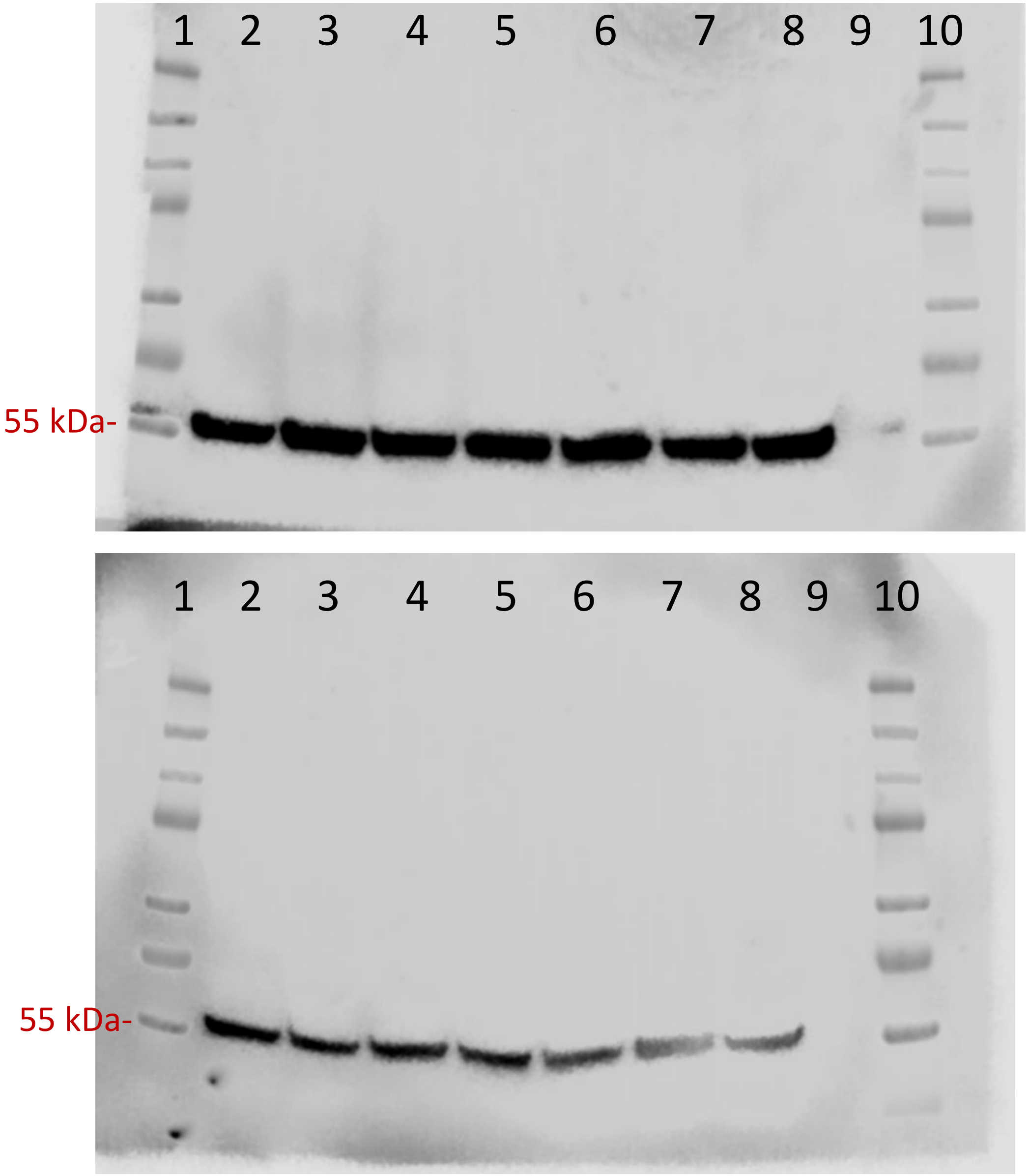

1. Protein marker
2. -zinc
3. +zinc
4. -zinc
5. +zinc
6. -zinc
7. +zinc
8. HCT116 lysate, not discussed
9. Blank
10. Protein marker

n=1 (lanes 2–3)
n=2 (lanes 4–5)
n=3 (lanes 6–7)

α/β tubulin Densitometry

	tubulin							
	Image Name	Channel	Name	Signal	Total	Area	Bkgnd.	Type
n=1	0000604_02	Chemi	00001	0.101968	0.169665	2145	3.16E-05	Signal
	0000604_02	Chemi	00002	0.070804	0.129999	2145	2.76E-05	Signal
n=2	0000604_02	Chemi	00003	0.087805	0.137475	2145	2.32E-05	Signal
	0000604_02	Chemi	00004	0.077943	0.122627	2145	2.08E-05	Signal
n=3	0000604_02	Chemi	00005	0.075227	0.100542	2145	1.18E-05	Signal
	0000604_02	Chemi	00007	0.052838	0.066135	2145	6.2E-06	Signal
hct116	0000604_02	Chemi	00006	0.059316	0.074914	2145	7.27E-06	Signal

Figure S1

β-catenin

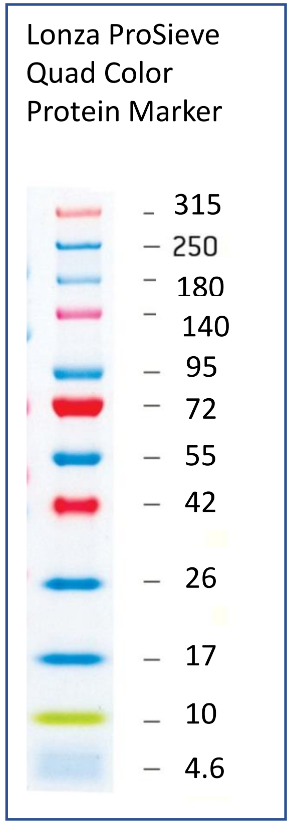

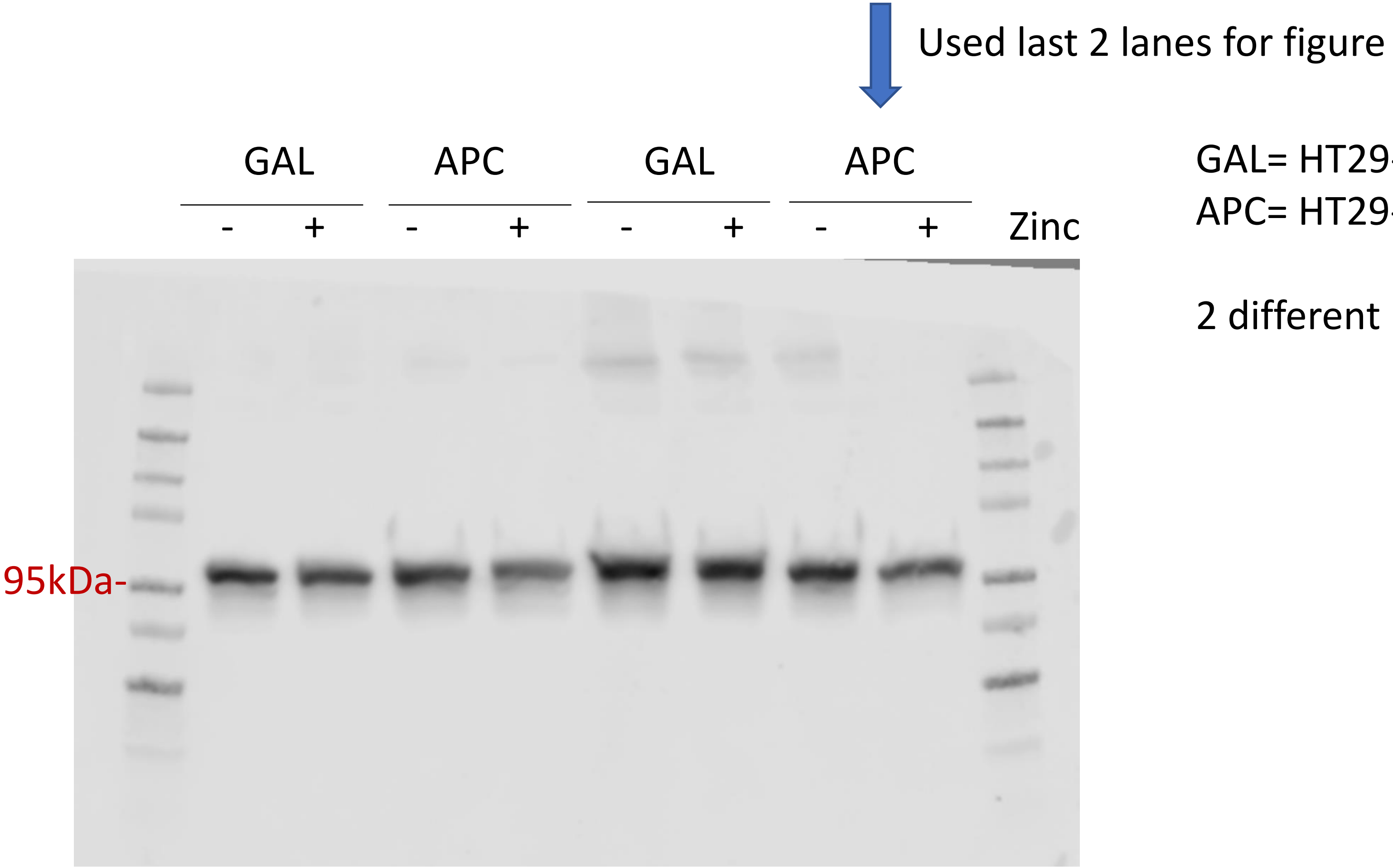

GAL= HT29-galactosidase cells
APC= HT29-wt-APC cells

2 different passages shown

Met

Lonza ProSieve Quad Color Protein Marker

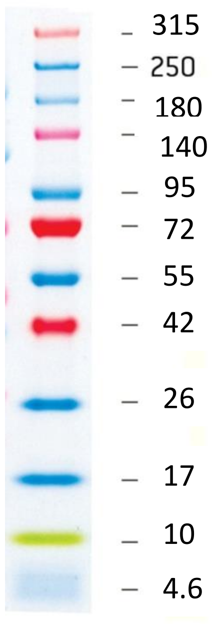

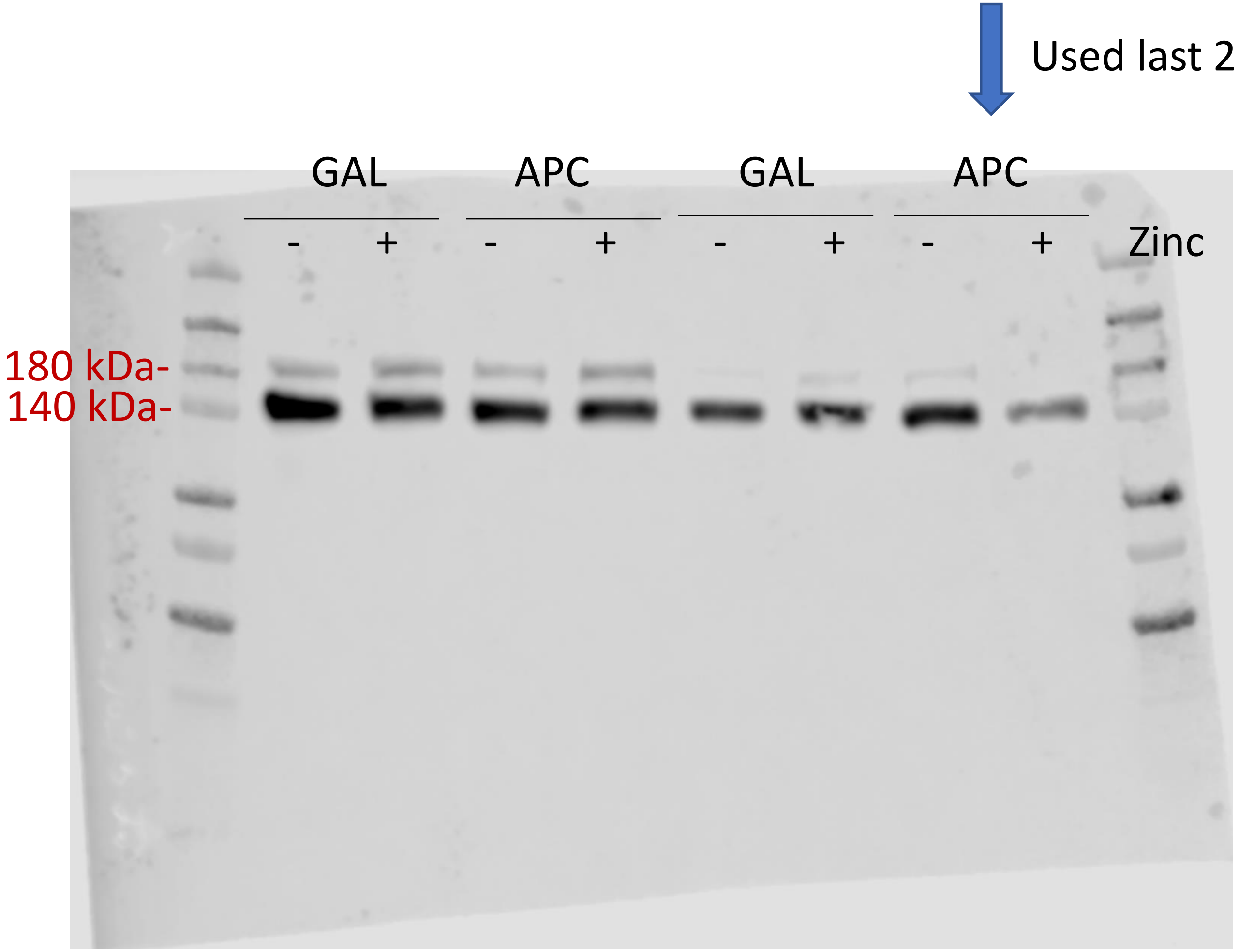

GAL= HT29-galactosidase cells
APC= HT29-wt-APC cells

2 different passages shown

c-Jun

Used last 2 lanes for figure

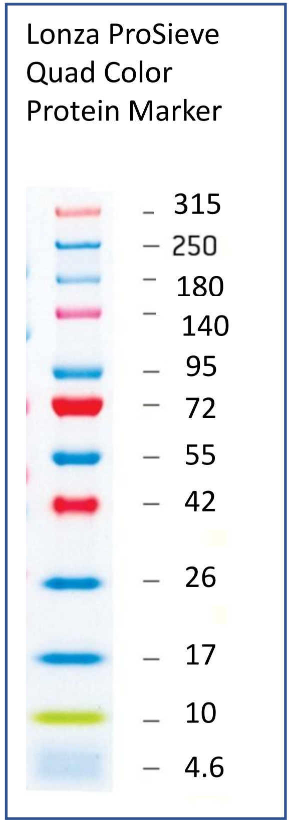

180 kDa-
140 kDa-
48 kDa-

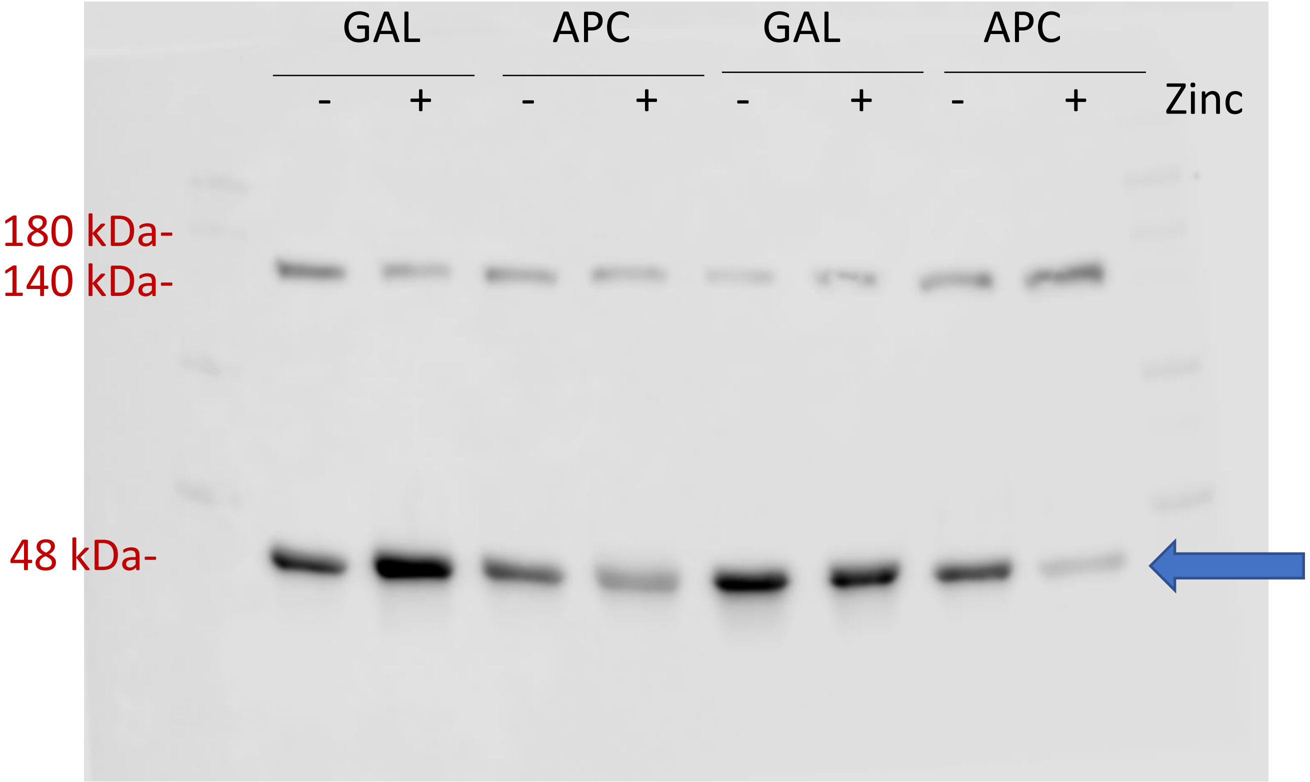

GAL= HT29-galactosidase cells
APC= HT29-wt-APC cells

2 different passages shown

CD44

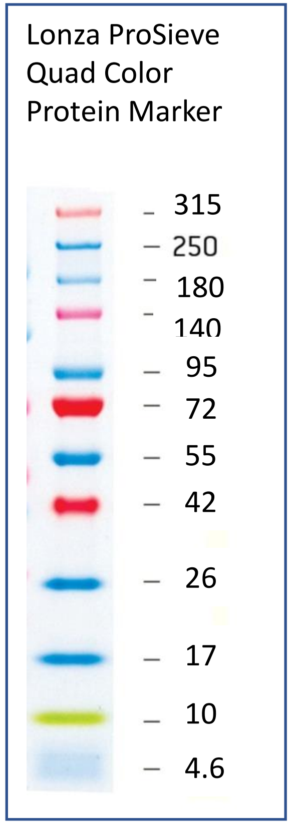

Used last 2 lanes for figure

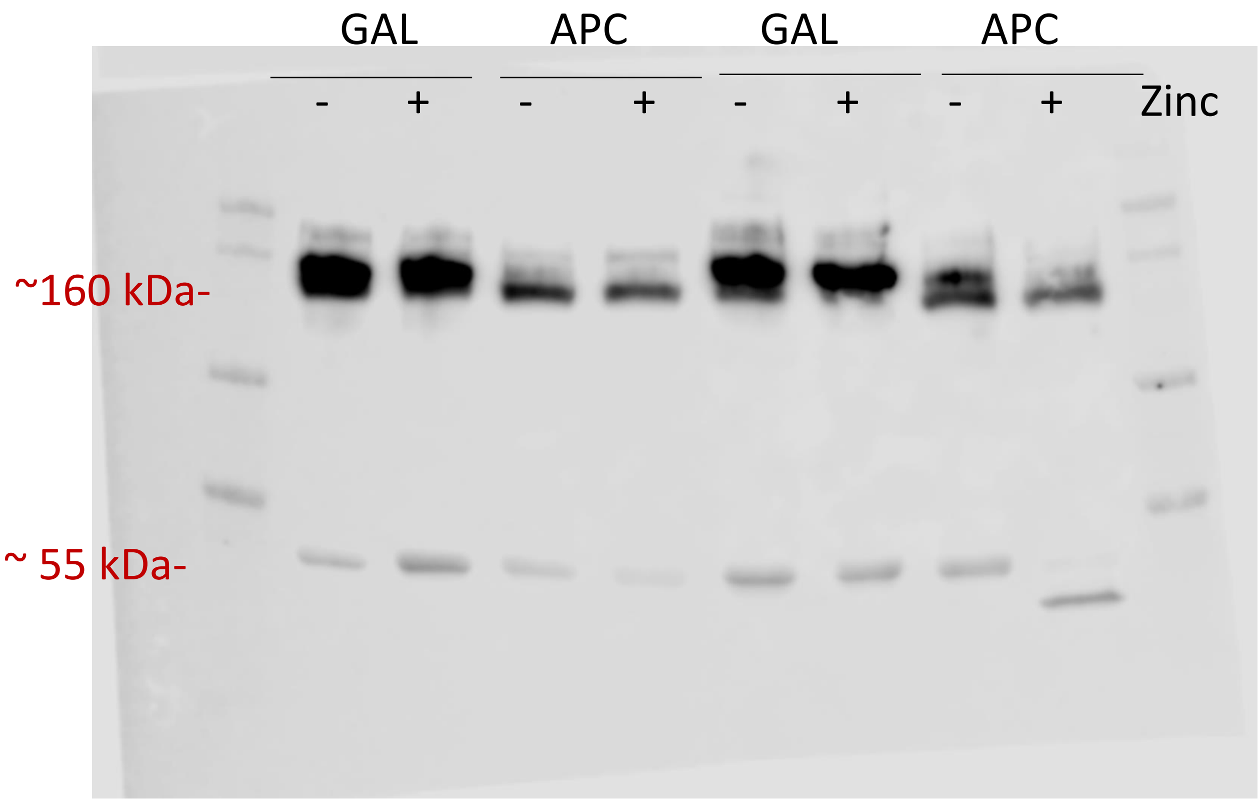

GAL= HT29-galactosidase cells
APC= HT29-wt-APC cells

2 different passages shown

c-Myc

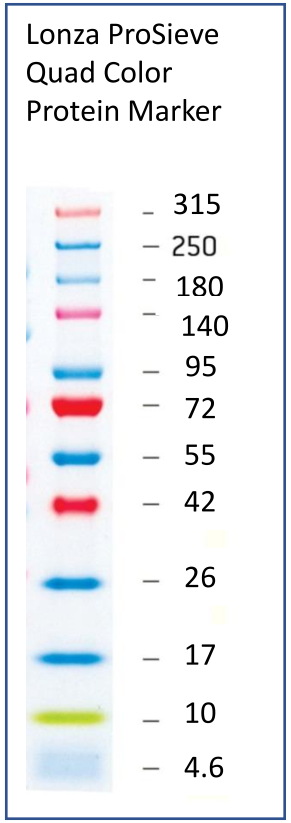

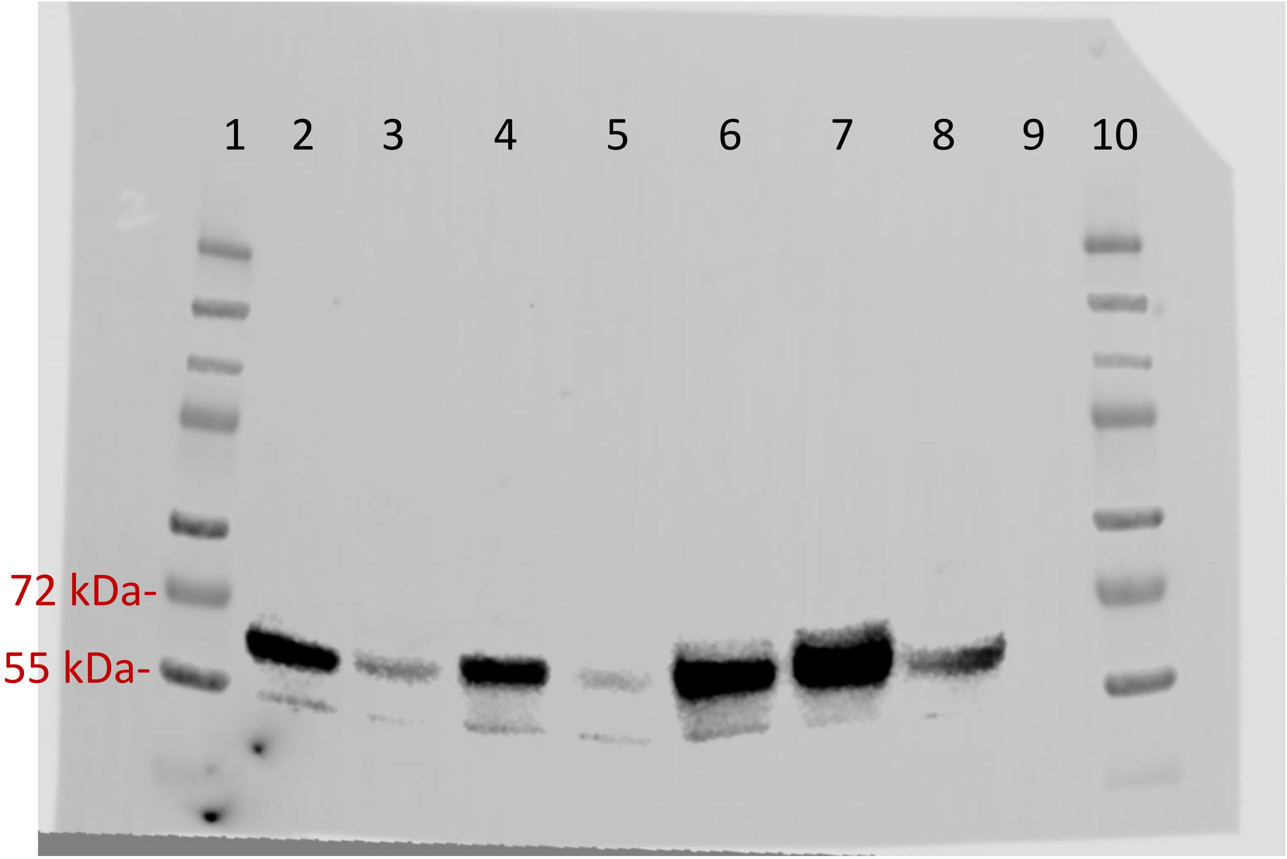

From series in Figure 5
Used n=2 for supplemental figure

1. Protein marker
2. -zinc
3. +zinc
   n=1
4. -zinc
5. +zinc
   n=2
6. -zinc
7. +zinc
   n=3
8. HCT116 lysate, not discussed
9. Blank
10. Protein marker

α/β- tubulin

Lonza ProSieve Quad Color Protein Marker

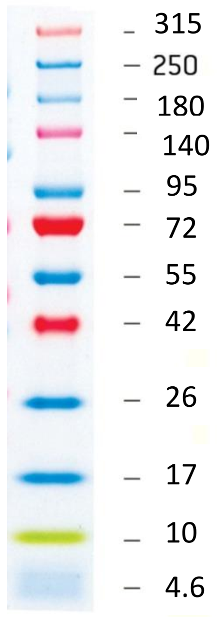

Used last 2 lanes for figure

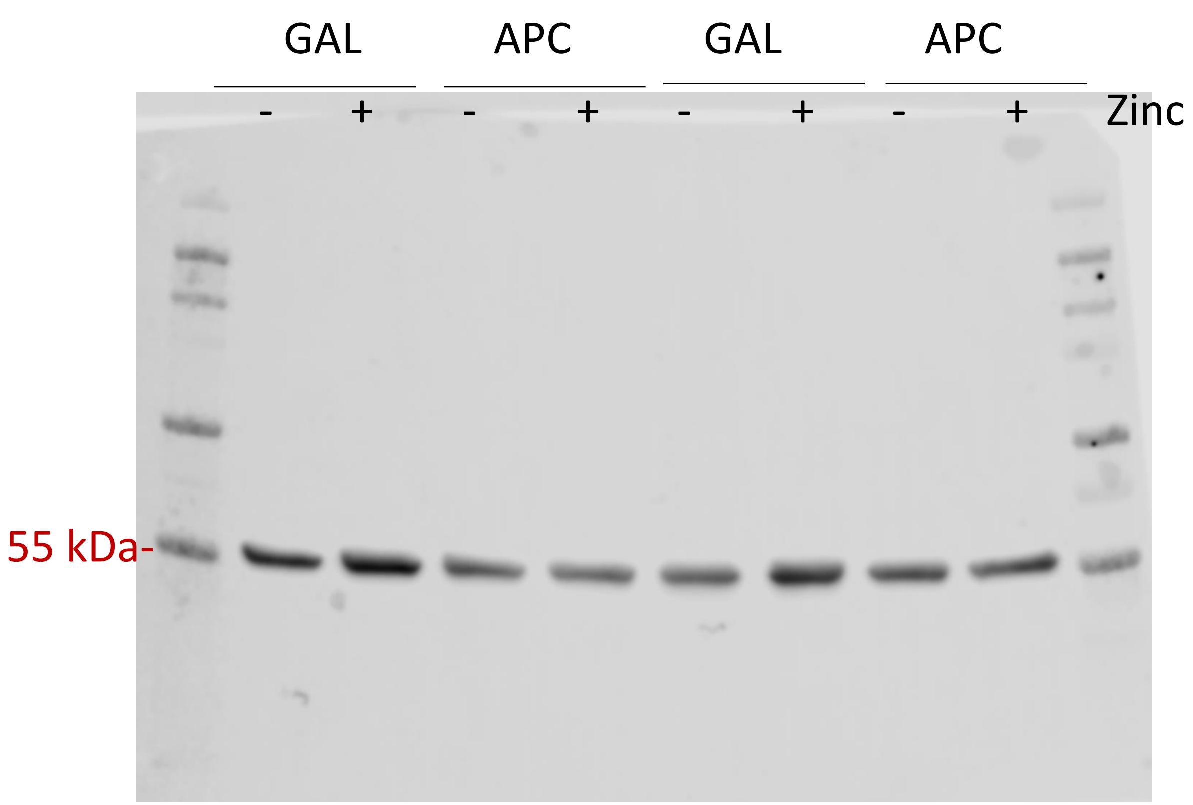

GAL= HT29-galactosidase cells
APC= HT29-wt-APC cells

2 different passages shown
